# Cine Displacement ENcoding imaging with Stimulated Echoes (cine-DENSE) confirms systolic myocardial dysfunction in asymptomatic patients with type 2 diabetes mellitus: comparison with MR-tagging

**DOI:** 10.1186/1532-429X-13-S1-P280

**Published:** 2011-02-02

**Authors:** Laura Ernande, Han Wen, Cyrille Bergerot, Hélène Thibault, Michel Ovize, Geneviève Derumeaux, Pierre Croisille

**Affiliations:** 1 Louis Pradel Hospital, Lyon, France; 2 National Heart, Lung, and Blood Institute, National Institutes of Health,, Bethesda, MD, USA

## Introduction

Diabetic cardiomyopathy contributes to increased cardiovascular mortality in diabetes mellitus (DM) patients and is characterized by a progressive alteration of left ventricular (LV) function. At a preclinical stage, a decrease in systolic myocardial strain has been suggested in echocardiographic studies.

MRI techniques remain the gold standard for quantification of myocardial deformation but only a single study suggested systolic abnormalities in type 2 DM patients with evidence of diastolic dysfunction.

MR-tagging is the most common technique for strain calculation using CMR but is intrinsically limited in measuring transmural variations. Cine-Displacement ENcoding Imaging with Stimulated Echoes(DENSE) has been recently proposed as an alternative that benefits from an increased spatial resolution.

## Purpose

To evaluate whether cine-DENSE and MR-tagging confirm the existence of a sub-clinical myocardial dysfunction in a population of type 2 DM patients with no sign or history of heart disease and normal conventional echo and MRI parameters.

## Methods

37 patients with type 2 DM (50.6±5.6 years, 8 females, HbA1c 7.6±1.2%) and 21 age-matched controls (49.7±8.0 years, 11 females) underwent a CMR study on a 1.5T scanner. Subjects were excluded if standard echocardiography showed significant abnormality. After a standard CMR study for conventional LV function assessment, two-dimensional cine-DENSE pulse sequence with short-echo train echo-planar imaging readout and cine-tagging with complementary spatial modulation of magnetization(CSPAMM) were acquired in short axis views at the same basal, mid and apical levels. LV volumes and ejection fraction were measured on cine-MRI images. Regional circumferential maximal systolic strain(ε_c_) was calculated from cine-DENSE and MR-tagging acquisitions on 16 LV segments. Average maximal systolic strain in each slice and a whole heart mean value(ε_c_mean) for each patient were calculated. Post-processing of cine-DENSE acquisitions included adaptive phase-unwrapping and spatial filtering. CSPAMM images were processed using *InTag* post-processing toolbox (Creatis, Lyon, France) implemented in OsiriX software (Geneva, Switzerland) with motion estimation based on the *Sine Wave Modeling* approach.

## Results

Standard cine-MRI LV function parameters were normal and comparable between groups (table 1). Whereas LV ejection fraction was similar in the 2 groups, cine-DENSE showed a significant decrease in ε_c_ at basal, mid and apical LV level and in ε_c_mean in the DM group as compared to controls. MR-tagging confirmed a decrease in ε_c_ at the 3 LV levels and in ε_c_mean in DM patients as compared with controls.

**Table 1:** Left ventricular function in type 2 diabetes mellitus patients and controls.

	DM patients	Controls	P
LVEDV (mL)	120 ± 26	129 ± 28	0.26
LVESV (mL)	41 ± 12	41 ± 12	0.90
LVEF (%)	66 ± 6	68 ± 6	0.30

ε_c_ base MR-tagging	-0.173 ± 0.040	-0.200 ± 0.028	0.004
ε_c_ mid MR-tagging	-0.177 ± 0.045	-0.220 ± 0.035	<0.001
ε_c_ apex MR-tagging	-0.189 ± 0.056	-0.232 ± 0.025	<0.001
ε_c_ mean MR-tagging	-0.179 ± 0.045	-0.216 ± 0.025	<0.001

ε_c_ base cine-DENSE	-0.134 ± 0.019	-0.155 ± 0.019	<0.001
ε_c_ mid cine-DENSE	-0.150 ± 0.021	-0.174 ± 0.020	<0.001
ε_c_ apex cine- DENSE	-0.153 ± 0.022	-0.193 ± 0.018	<0.001
ε_c_ mean cine-DENSE	-0.144 ± 0.016	-0.171 ± 0.016	<0.001

LVEDV= left ventricular end-diastolic volume; LVESV= left ventricular end-systolic volume; LVEF= left ventricular ejection fraction; ε_c_ =Régional circumferential maximal systolic strain.

## Conclusions

Cine-DENSE and MR-tagging confirm subclinical myocardial dysfunction in asymptomatic patients with Type II DM.

